# Supplementary material for: Identification of a novel potassium channel (GiK) as a potential drug target in Giardia lamblia: Computational descriptions of binding sites
Source: PeerJ. 2019 Feb 27;7:e6430. doi: 10.7717/peerj.6430 (PMC6397635; doi:10.7717/peerj.6430)
Supplement: Supplemental Information 1 — A total of 51 potassium channel sequences from genomes of different species, deposited in the NCBI protein database (http://www.ncbi.nlm.nih.gov/protein), were compared by BLAST algorithm with the Giardia genome database (http://giardiadb.org/giardiadb/). [file peerj-07-6430-s001.docx]

| Accession number | Organism | Accession number | Organism |
| --- | --- | --- | --- |
| WP_022541369.1 | *Aeropyrum camini* | **NP_001245037.1** | *Macaca mulatta* |
| AA87294.1 | *Arabidopsis thaliana* | **NP_032444.2** | *Mus musculus* |
| AAA96810.1 | *Arabidopsis thaliana* | **WP_006090946.1** | *Natronorubrum tibetense* |
| WP_007053655.1 | *Bifidobacterium longum* | **BAD29689.1** | *Oryza sativa Japonica Group* |
| AEE68730.1 | *Bordetella pertussis* | **XP_001419910.1** | *Ostreococcus lucimarinus* |
| YP_001776865.1 | *Burkholderia cenocepacia* | **WP_006207013.1** | *Paenibacillus vortex* |
| T34116 | *Caenorhabditis elegans* | **CDM31621.1** | *Penicillium roqueforti* FM164 |
| YP_008796598.1 | *Candidatus Caldiarchaeum subterraneum* | **ABY86891.1** | *Populus euphratica* |
| XP_005844251.1 | *Chlorella variabilis* | **ACB56631.1** | *Populus euphratica* |
| XP_006421368.1 | *Citrus clementina* | **XP_006371939** | *Populus trichocarpa.1* |
| XP_006451007.1 | *Citrus clementina* | **XP_006373346.1** | *Populus trichocarpa* |
| XP_006486795.1 | *Citrus sinensis* | **XP_007383667.1** | *Punctularia strigosozonata* |
| CDS37581.1 | *Echinococcus multilocularis* | **XP_002511827.1** | *Ricinus communis* |
| YP_002407586.1 | *Escherichia coli* IAI39 | **WP_006887331.1** | *Rothia aeria* |
| WP_024212520.1 | *Escherichia spp* | **301 NP_707157.2** | *Shigella flexneri 2a str.* |
| AF197333_1 | *Eucalyptus camaldulensis* | **CAA56175.1** | *Solanum tuberosum* |
| AAP94028.1 | *Gallus gallus* | **XP_006359367.1** | *Solanum tuberosum* |
| XP_003529954.1 | *Glycine max* | **NP_631700.1** | *Streptomyces coelicolor* |
| YP_656932.1 | *Haloquadratum walsby* | **EJ3 ACS32631.1** | *Thermococcus_gammatolerans* |
| NP_002223.3 | *Homo sapiens* | **WP_003043765.1** | *Thermus aquaticus* |
| AAA61276.1 | *Homo sapiens* | **CDW52461.1** | *Trichuris trichiura* |
| BAK03046.1 | *Hordeum vulgare subsp. vulgare* | **WP_033004452.1** | *Vibrio azureus* |
| CDS30290.1 | *Hymenolepis microstoma* | **CAN69248.1** | *Vitis vinifera* |
| AEO96823.2 | *Lateolabrax japonicus* | **AR21353.1** | *Zea mays* |
| CCQ21618.1 | *Listeria monocytogenes* | **DAA62579.1** | *Zea mays* |
| AFK47677.1 | *Lotus japonicus* |  |  |
