# Supplementary material for: Identification of a novel potassium channel (GiK) as a potential drug target in Giardia lamblia: Computational descriptions of binding sites
Source: PeerJ. 2019 Feb 27;7:e6430. doi: 10.7717/peerj.6430 (PMC6397635; doi:10.7717/peerj.6430)
Supplement: Supplemental Information 2 — A total of 290 compounds were docked. [file peerj-07-6430-s002.docx]

| **Compounds** | | | | |
| --- | --- | --- | --- | --- |
| UCL_1684 | Amitriptyline | Diazoxide | ZINC13442202 | ZINC13760169 |
| ZINC38144725 | Dequalinium | E_4031 | ZINC13489801 | ZINC13760170 |
| Terfenadine | ZINC01539875 | Haloperidol | ZINC13489802 | ZINC13760171 |
| ZINC00018512 | ZINC13489789 | Hydrochlorothiazide | ZINC13489805 | ZINC13760172 |
| ZINC00598948 | Quinidine | Hydroflumethiazide | ZINC13489808 | ZINC13760173 |
| Bicuculine | ZINC00014006 | Ibutilide | ZINC13489809 | ZINC13760182 |
| Cromoglicic acid | ZINC01535218 | MK_499 | ZINC13489811 | ZINC13760195 |
| Penitrem_A | ZINC13760206 | Niflumic_Acid | ZINC13489812 | ZINC13760196 |
| BMS_204352 | ZINC27617400 | NS004 | ZINC13489815 | ZINC13760210 |
| NS1643 | Psora_4 | NS1619 | ZINC13489816 | ZINC13760233 |
| Paxilline | ZINC18189761 | NS309 | ZINC13489821 | ZINC13760234 |
| CP_339818 | Pimaric_acid | RPR260243 | ZINC13489822 | ZINC13760235 |
| Tubocurarine | Miconazole | ZINC00008767 | ZINC13489824 | ZINC13760236 |
| ZINC13489797 | ZINC13760204 | ZINC00009347 | ZINC13489825 | ZINC13760237 |
| UK_78282 | ZINC13760205 | ZINC00025912 | ZINC13489826 | ZINC13760242 |
| Verruculogen | ZINC13760213 | ZINC00025913 | ZINC13489827 | ZINC13760243 |
| ZINC13489806 | Flecainide | ZINC00025918 | ZINC13489831 | ZINC13760244 |
| ZINC13644028 | Mepivacaine | ZINC00537795 | ZINC13491157 | ZINC13760245 |
| DIDS | ZINC13489786 | ZINC00537805 | ZINC13491160 | ZINC13760246 |
| ZINC01535217 | ZINC13760202 | ZINC00588923 | ZINC13491161 | ZINC13905994 |
| ZINC13442159 | ZINC13777065 | ZINC00591407 | ZINC13491174 | ZINC13905995 |
| ZINC38144724 | 1-Ethyl-2-Benzimidazolinone | ZINC00591408 | ZINC13524640 | ZINC13905996 |
| Bicuculline methiodide | ZINC13760207 | ZINC01535221 | ZINC13524661 | ZINC13905997 |
| ZINC13489814 | ZINC13760214 | ZINC01535692 | ZINC13524693 | ZINC13905998 |
| ZINC13489817 | ZINC03935230 | ZINC01539874 | ZINC13589603 | ZINC13905999 |
| ZINC00015850 | ZINC13557606 | ZINC01540211 | ZINC13589604 | ZINC14177134 |
| ZINC00603820 | ZINC13777062 | ZINC01540212 | ZINC13644007 | ZINC19800352 |
| ZINC01539867 | ZINC27617403 | ZINC01540213 | ZINC13760128 | ZINC26845790 |
| ZINC13489795 | Dofetilide | ZINC01540214 | ZINC13760129 | ZINC27880082 |
| ZINC13489796 | Retigabine | ZINC01540215 | ZINC13760130 | ZINC27880088 |
| ZINC13489807 | ZINC00005768 | ZINC01540216 | ZINC13760131 | ZINC29239260 |
| ZINC13489823 | ZINC13760203 | ZINC03808605 | ZINC13760132 | ZINC29239267 |
| ZINC29309163 | ZINC13777063 | ZINC03872277 | ZINC13760133 | ZINC29239274 |
| Niguldipine | ZINC13777067 | ZINC03940690 | ZINC13760134 | ZINC29239280 |
| ZINC13489799 | Correolide | ZINC03940691 | ZINC13760135 | ZINC29239288 |
| XE991 | ZINC03935234 | ZINC03995738 | ZINC13760136 | ZINC29239296 |
| ZINC01539870 | ZINC03935235 | ZINC03995739 | ZINC13760137 | ZINC29239301 |
| ZINC13489790 | ZINC03946466 | ZINC13442136 | ZINC13760138 | ZINC29239307 |
| Imipramine | ZINC13777069 | ZINC13442138 | ZINC13760139 | ZINC29246194 |
| Trifluoroperazine | ZINC13777072 | ZINC13442140 | ZINC13760140 | ZINC29246201 |
| ZINC13489791 | Procaine | ZINC13442142 | ZINC13760141 | ZINC29248689 |
| ZINC13489800 | Zoxazolamine | ZINC13442144 | ZINC13760142 | ZINC29248697 |
| ZINC13489804 | ZINC13777058 | ZINC13442145 | ZINC13760143 | ZINC29248704 |
| ZINC13489830 | ZINC18096411 | ZINC13442147 | ZINC13760146 | ZINC29248710 |
| ZINC13760212 | ZINC13777075 | ZINC13442148 | ZINC13760147 | ZINC29309752 |
| Linopirdine | ZINC13643922 | ZINC13442150 | ZINC13760148 | ZINC29309758 |
| ZINC13442157 | Chlorzoxazone | ZINC13442152 | ZINC13760149 | ZINC29309775 |
| ZINC13489810 | ZINC13579814 | ZINC13442154 | ZINC13760154 | ZINC29309786 |
| ZINC13489818 | LY_97241 | ZINC13442155 | ZINC13760155 | ZINC29309804 |
| ZINC13489829 | Clofilium | ZINC13442165 | ZINC13760156 | ZINC29309831 |
| ZINC13489785 | Halothane | ZINC13442167 | ZINC13760157 | ZINC29473620 |
| TRAM_34 | 4_Aminopyridine | ZINC13442168 | ZINC13760162 | ZINC35942034 |
| ZINC13489794 | TEA | ZINC13442170 | ZINC13760163 | ZINC35965574 |
| ZINC13489798 | AM_92016 | ZINC13442172 | ZINC13760164 | ZINC38152038 |
| ZINC13489784 | Astemizole | ZINC13442173 | ZINC13760165 | ZINC38157782 |
| ZINC13489803 | Bendroflumethiazide | ZINC13442195 | ZINC13760166 | ZINC40670687 |
| ZINC13489813 | CGS7184 | ZINC13442198 | ZINC13760167 | ZINC40670690 |
| ZINC13557604 | Chromanol_293B | ZINC13442200 | ZINC13760168 | ZINC45257896 |
